# A Bioinformatics Approach to Identifying Potential Biomarkers for *Cryptosporidium parvum*: A Coccidian Parasite Associated with Fetal Diarrhea

**DOI:** 10.3390/vaccines9121427

**Published:** 2021-12-02

**Authors:** Mumdooh J. Sabir, Ross Low, Neil Hall, Majid Rasool Kamli, Md. Zubbair Malik

**Affiliations:** 1 Norwich Medical School, Faculty of Medicine and Health Sciences, University of East Anglia, Norwich NR4 7TJ, UK; mumdooh-jamal-s.sabir@uea.ac.uk; 2 Department of Computer Science, Faculty of Computing and Information Technology, King Abdulaziz University, Jeddah 21589, Saudi Arabia; 3 Earlham Institute, Norwich Research Park, Norwich NR4 7UZ, UK; ross.low@earlham.ac.uk (R.L.); neil.hall@earlham.ac.uk (N.H.); 4 Centre of Excellence in Bionanoscience Research, King Abdulaziz University, Jeddah 21589, Saudi Arabia; 5 Department of Biological Sciences, King AbdulAziz University, Jeddah 21589, Saudi Arabia; 6 School of Computational and Integrative Science, Jawaharlal Nehru University, New Delhi 110067, India

**Keywords:** *Cryptosporidium parvum* infection, Differentially Expressed Genes (DEGs), key protein, miRNA, gene ontology

## Abstract

*Cryptosporidium parvum (C. parvum)* is a protozoan parasite known for cryptosporidiosis in pre-weaned calves. Animals and patients with immunosuppression are at risk of developing the disease, which can cause potentially fatal diarrhoea. The present study aimed to construct a network biology framework based on the differentially expressed genes (DEGs) of *C. parvum* infected subjects. In this way, the gene expression profiling analysis of *C. parvum* infected individuals can give us a snapshot of actively expressed genes and transcripts under infection conditions. In the present study, we have analyzed microarray data sets and compared the gene expression profiles of the patients with the different data sets of the healthy control. Using a network medicine approach to identify the most influential genes in the gene interaction network, we uncovered essential genes and pathways related to *C. parvum* infection. We identified 164 differentially expressed genes (109 up- and 54 down-regulated DEGs) and allocated them to pathway and gene set enrichment analysis. The results underpin the identification of seven significant hub genes with high centrality values: ISG15, MX1, IFI44L, STAT1, IFIT1, OAS1, IFIT3, RSAD2, IFITM1, and IFI44. These genes are associated with diverse biological processes not limited to host interaction, type 1 interferon production, or response to IL-gamma. Furthermore, four genes (IFI44, IFIT3, IFITM1, and MX1) were also discovered to be involved in innate immunity, inflammation, apoptosis, phosphorylation, cell proliferation, and cell signaling. In conclusion, these results reinforce the development and implementation of tools based on gene profiles to identify and treat Cryptosporidium parvum-related diseases at an early stage.

## 1. Introduction

Cryptosporidium species are generally widespread protozoan parasites that cause severe gastrointestinal problems in animals and humans [1]. *Cryptosporidium parvum* infects a variety of domestic and wild animals, in addition to humans, it is known to be an important agent associated with zoonotic cryptosporidiosis [2]. Calves under the age of 8 weeks are the most frequent victims of this Cryptosporidium species, often associated with acute diarrhoea, morbidity, and death [3]. Animals infected with zoonotic subtypes could potentially spread the disease to other animals, farmers, and humans. Pre-weaned calves have occasionally been found to host other Cryptosporidium species [3,4].

The Centers for disease Control and Prevention, United States, have classified *Cryptosporidium* as an emerging protozoan parasite affecting 748,000 humans in the United States every year [5]. Currently, about 40 *Cryptosporidium* species have been detected that can infect vertebrates [6]. The mode of infection primarily involves the infection of the microvilli of the small intestine leading to disruption of the ionic balance in the intestinal tract, resulting in gastroenteritis. The gastrointestinal tract hosts the intestinal microbiota as a complex organ. However, the gastrointestinal epithelium also acts as an active site of infection in many vertebrate hosts; therefore, understanding the host-parasite interaction in Cryptosporidium has significant importance [7,8].

Cryptosporidium species are identified using genotyping techniques such as RFLP, PCR, and the Sanger sequence for 18S rRNA gene [9,10]. In addition, the gp60 gene is frequently used for genome sequencing in *C. parvum* and *Cryptosporidium hominis* (*C. hominis*). Depending on the species involved, the host age and immune condition, *C. parvum* is the most common parasite that causes Cryptosporidiosis [11]. People in under-equipped cities in underdeveloped nations succumb to enteric infections through contaminated food and drinking water owing to infectious oocysts in faeces, resulting in diarrhoea [12,13]. Infected hosts shed many oocytes that are resistant to disinfectants like chlorine and can withstand routine water treatment. Observations indicate that even fewer than 10 *Cryptosporidium* oocysts are necessary to cause illness [14,15]. Therefore, the study of miRNAs at the host-pathogen interface is anticipated to yield helpful information for identifying possible molecular treatment targets for modulating *C. parvum* pathogenesis. We have performed a gene-transition study of differentially expressed gene (DEG) data and text mining between a *C. parvum* infected person and a healthy one. It is widely thought that, to know the function of a gene, it must be analyzed in the context of gene interaction network, because gene networks are commonly interpreted as encoding functional information in their connections. So, our study mainly focused on the “guilt-by-association (GBA)” presumption which states that physically and functionally linked genes are possibly participating in the same biological pathways, having comparable effects on the phenotypes [16].

After Cryptosporidium infection by targeting PDCD4, a pro-inflammatory protein that further increases NF-B activation in the biliary epithelial cell, TLR4/NF-B signaling is suppressed to some extent [17]. TLR4 expression in cholangiocytes is regulated by let-7i, which contributes to epithelial immunological responses against *C. parvum* infection [18]. Let-7i expression is reduced in cholangiocytes infected with *C. parvum* via a NFkB/MyD88 dependent pathway [18]. Consequently, using microarrays and bioinformatics the current study was carried out at the DEG and miRNA expression profiles of intestinal epithelial cells, which led to a better understanding of the immunologic features in *C. parvum*. Hence, gene expression biomarkers from infected tissue (intestinal epithelial cells) were investigated to find potential immune biomarkers for *C. parvum* infection and to uncover prospective immunological biomarkers.

Furthermore, the tissue had accumulated a pool of immune cells travelling from active disease sites towards lymphoid organs, so this tissue is useful for exploration. In *C. parvum* infected patients, interaction networks and validated relationship data were observed using curated and empirically identified differentially expressed genes. The study considers the network’s hubs, motifs, and modules equally when identifying biomarkers and regulatory pathways, rather than establishing a connection between them in gene-disease associations

## 2. Method and Material

**Figure 1:**
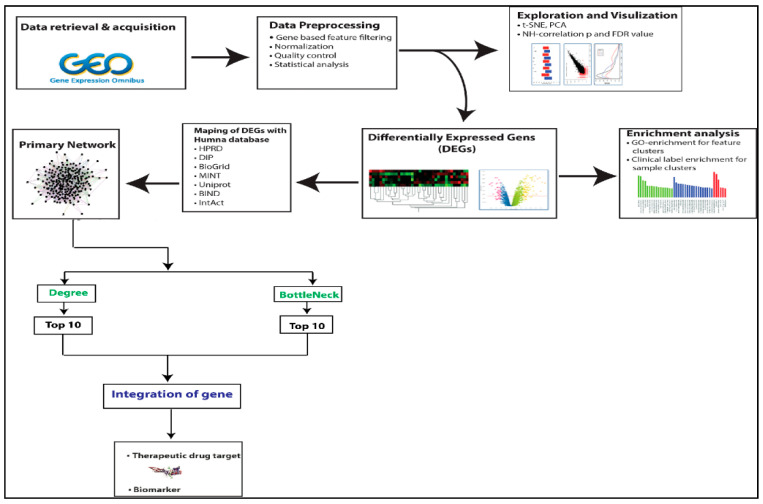
The workflow of the methodologies used in the study of the *C. parvum* infection-related PPI network is depicted in this diagram.

A detailed workflow is given in Figure 1.

### 2.1. Microarray Datasets Collection and Pre-Processing

GEO is a data archive repository for microarrays, chips, and high-throughput gene expression data [19]. The GEO (Affymetrix GPL17077 platform, Agilent-039494 Sure Print G3 Human GE v2 8x60K Microarray 039381 039381 (Probe Name version) was used to retrieve the gene expression dataset GSE87047 [20,21]. The probes were converted into their corresponding gene samples via the annotation interface. In the GSE87047dataset, 03 *C. parvum* infection intestinal epithelium tissue samples and 03 non-*C. parvum* infection samples were included. A large sample size could reliably expose differentially expressed genes (DEGs) or non-coding RNAs. Hence, GEO datasets with at least ten samples were chosen for further analysis [22]. To assure unbiased and deregulated gene expression data, the R-affy and Lumi packages’ RMA was used to perform background data correction and data standardization. The RMA approach was applied to reduce inconsistencies caused by the normalizing of individual Affymetrix GSE series, which conducts quintile normalization, since its precise differential change detection and constant variance on a log scale. When calculating fold-change to discover DEGs, the RMA method provides strong specificity and sensitivity. We also used the Bioconductor Package (Lumi pipeline), designed exclusively for Illumine data analysis (BeadChip). It verifies data consistency and normalization while also lowering data volatility [23,24].

### 2.2. Identification of Differentially Expressed Genes (DEGs)

We used R’s linear model for the microarray analysis (LIMMA) package to investigate DEGs in each GEO dataset, by doing simple *t*-test, moderate *t*-test, and f-test calculations. Even with fewer arrays, we can get consistent and reliable results by using the Empirical Bayes technique and lowering the standard errors. The DEGs between healthy and *C. parvum* infection were determined using the limma program [25]. DEGs are defined as genes with p<0.05, |log2 fold change|≥2 defined as upregulated DEGs and downregulated DEGs, respectively.

We used the LIMMA package in R to look into DEGs in each GEO dataset, and analyzed them by simple, moderate, and *f*-test’s. Further, with the application of Empirical Bayes and decreasing standard errors, it is possible to achieve steady and reliable results even with fewer arrays. The DEGs between healthy and *C. parvum* infection were determined using the limma program [25].

### 2.3. Construction and Analysis of PPI Networks

Specific DEGs identified following enrichment analysis were used for construction and analysis of PPI networks. The DEGs’ primary PPI network was created using the simple concept of one gene correspondence with one protein. The network was built using interaction data from the GeneMANIA database [26], and the file was checked and uploaded for further literature verification in Cytoscape [27,28]. The PPI networks were constructed in Cytoscape, and MCODE was used to identify the most critical module in the PPI networks.

### 2.4. Topological Properties of the Network

The network’s topological features are the first and most crucial foundational analysis—topological analysis aids in comprehending a network’s structure, making the underlying mechanisms easier to understand. The topological features of the PPI network of DEGs are determined by utilizing the Cytoscape plugins Network Analyzer [29] and Cytohubba [30] to quantify degree (*k*), betweenness centrality (CB), and bottleNeck (BN). The network features were examined to determine the network’s essential behaviours [31,32,33,34,35,36].

Degree(k)

A node/gene in the network determines the total number of links, expressed by the degree k. In the network regulation process, it determines a node’s local significance. The nodes are symbolized by N, whereas the edges are designated by E in the graph *G = (N, E)*. The degree of the $i^{th}$ node ($k_{i}$) is defined as $k_{i}=\sum_{ij}^{N}A_{ij}$, where $A_{ij}$ denotes the graph’s adjacency matrix elements.

Betweenness

The measure of a node’s proportion of all shortest-path traffic from all feasible routes from nodes I to j is called betweenness centrality. As a result, it is a metric that describes a node’s ability to profit from the flow of information throughout the network [37] and its ability to affect signal processing across the network’s other nodes [38]. $C_{B}\left(v\right)$ of node, v may be calculated using Equation (1) if $d_{ij}\left(v\right)$ indicates the number of geodesic pathways from one node I with another node j passing through the node v.

(1) $$C_{b}\left(v\right)=\underset{i,j;i\neq j\neq k}{\sum }\frac{d_{ij}\left(v\right)}{d_{ij}}$$

Equation (2) summarizes the normalized betweenness centrality, where *M* denotes the number of node pairs excluding *v*.

(2) $$C_{B}\left(v\right)=\frac{1}{MC_{b}\left(v\right)}$$

BottleNeck (BN)

The high betweenness nodes are the bottleneck. It can be estimated using central betweenness, which seems to be a measurement of the centrality of a node in a network, and equal to the number of shortest paths. Let D n be the shortest node-rooted path tree,

(3) $$\mathrm{BN}\left(v\right)=\sum x\in {\mathrm{VR}}_{n}\left(v\right)$$

where $R_{n}\left(v\right)=1$ if more than $\frac{V(D_{n})}{4}$ paths from node n to other nodes in $D_{n}$ meet at the vertex v; otherwise $R_{n}\left(v\right)=0$.

### 2.5. Identification of Biomarker

Centrality measurements are used to identify potential therapeutical target genes since they can characterize the most impacting genes in a complex network, capable of quick information transmission, reception, and sensitivity to local and global perturbations. We calculated the centrality score for each gene in the *C. parvum* infection network using cytohubba [30] and network analyzer [29] for each centrality degree and bottleNeck. A gene with a higher degree (k) and bottleneck (BN) value can help identify the biological entity in the network that plays the most critical role. So, we used cytoHubba and network analyzer to measure degree (k) and BN. First, we chose the top 10 ranking genes based on degree and BN. Then, in the degree and BN of the network, we discovered the common genes. We found biomarkers in intestinal tissue with extremely high hub and BNvalues, indicating that these genes play a significant regulatory function in the *C. parvum* infection network.

### 2.6. Identification of miRNA Associated with Hub Genes

MIENTURNET (MicroRNA ENrichment TURned NETwork) was used for microRNA target enrichment analysis. The tool is based on miRTarBase and TargetScan sequence-based miRNA target predictions [39]. Screening of miRNA of hub genes and driver genes was performed using *MIENTURNET*. The significant functional enrichment of predicted miRNA was performed using the MIENTURNET tool. Using Cytoscape 3.6.1, we constructed a hub genes-miRNA interaction co-expression network. Disease ontology (human), REACTOME and KEGG, were also used to perform functional enrichment analysis between target genes and identified miRNAs.

## 3. Results

### 3.1. Extraction and Pre-Processing of Microarray Data

**Figure 2:**
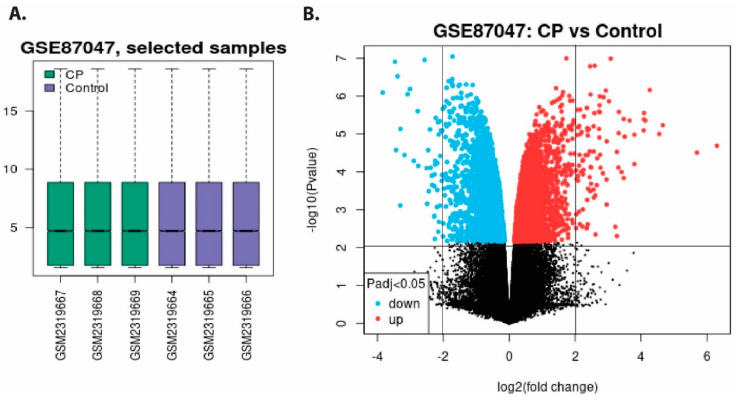
(**A**) Normalized boxplots of microarray dataset of GSE87047. Green boxplots represent *C. parvum*-infected tissue samples (GSM2319667, GSM2319668, and GSM2319669) and blue boxplots show healthy samples (GSM2319664, GSM2319665, and GSM2319666). (**B**) The volcano plot displays differentially expressed downregulated and upregulated genes in infected and uninfected hosts. Cyan scatters represent DEGs that are downregulated, red scatters indicate DEGs that are upregulated, and black scatters indicate DEGs that are not significant. The *x*-axis displays the log2 fold change, and *y*-axis depicts −log10 fold change (*p* values).

According to the methodology section’s inclusion and exclusion criteria, microarray gene expression profiles with accession number GSE870476613 were selected containing expression data from intestinal epithelial tissue infected with *C. parvum* infection (Table S1). To identify DEGs between *C. parvum* infected patient and healthy control, we used GSE87047 datasets. The volcano plot and box plot of healthy and *C. parvum* infection for tissue samples are shown in Figure 2A. We found 163 DEGs, of which 109 up and 54 down-regulated DEGs from the samples of *C. parvum* infected tissue, and healthy control sets were compared after applying the statistical threshold of log2 (fold change) BH-p-value.

### 3.2. Functional and Pathway Enrichment Analysis

**Figure 3:**
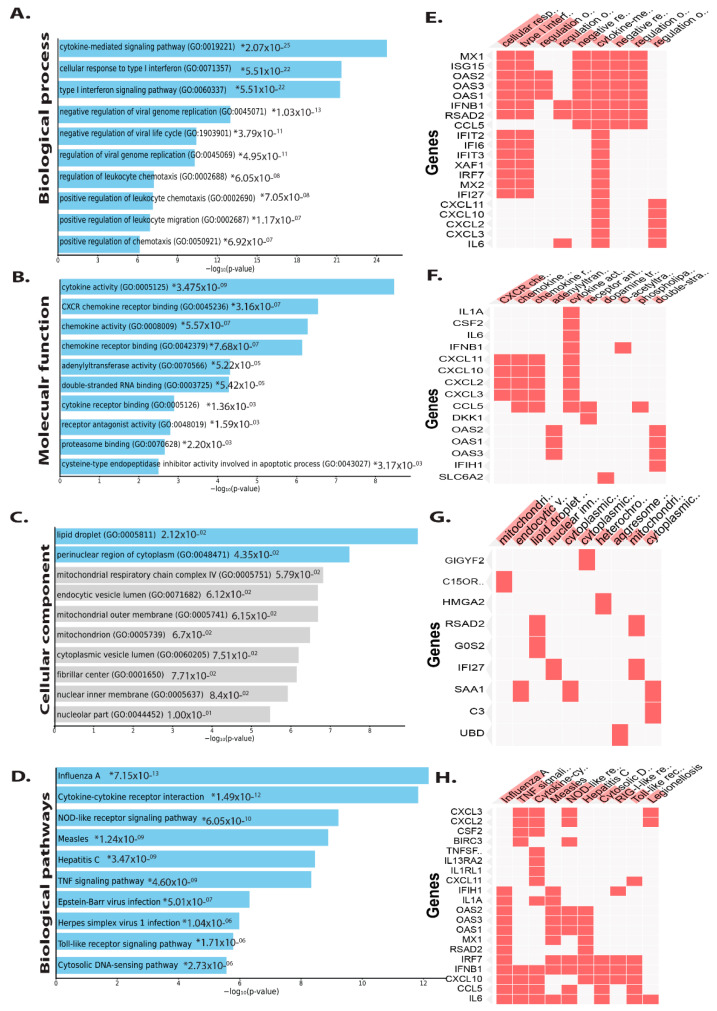
DEGs that are upregulated are classified using Gene Ontology. Plots of the log-*p* value and the enriched Enrichr-based combination score. The *p*-value indicates the significance of enrichment and the number of genes/proteins in the category. Clustergram generated by Enrichr using upregulated genes. Genes associated with each term are indicated by red cells in the matrix, with *p*-values showing the top 10 most enriched terms <0.05 (**A**) Biological process (**B**) Molecular function (**C**) Cellular component and (**D**) Biological pathway. (**E**) In clustergram, the red cells in the matrix indicate the genes associated with the top 10 significant biological processes. (**F**) In clustergram, throughout the matrix, red cells represent the genes involved with the top 10 significant molecular functions. (**G**) In clustergram, the red cells in the matrix indicate the genes associated with the top 10 important cellular component. (**H**) In clustergram, throughout the matrix, red cells represent the genes involved with the top 10 significant biological pathways. * denotes the highly significant (*p* value).

**Figure 4:**
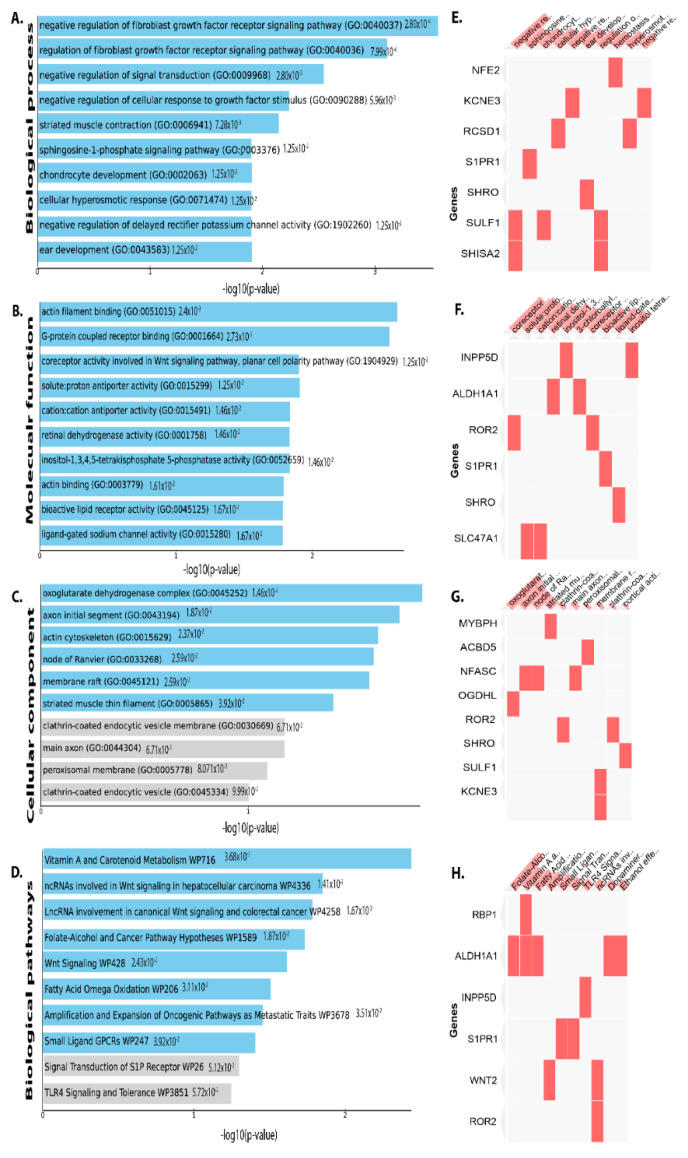
DEGs that have been downregulated are classified using gene ontology. Plots of the log-*p* value and the enriched enrichr-based combination score. The *p* value indicates the significance of enrichment and the number of genes/proteins in the category. Using downregulated genes, Clustergram was generated by Enrichr. Genes associated with each term are indicated by the red cells in the matrix, depicting top 10 enriched terms with *p*-value < 0.05 (**A**) Biological process (**B**) Molecular function (**C**) Cellular component and (**D**) Biological pathway. (**E**) Clustergrams display the genes associated with the top 10 biological processes in red cells in a matrix. (**F**) In clustergram, the red cells in the matrix indicate the genes associated with the top 10 significant molecular function. (**G**) In clustergram, the red cells in the matrix indicate the genes associated with the top 10 important cellular components. (**H**) Clustergrams display the genes related to the top 10 biological pathways in red cells in a matrix.

GO enrichment analysis was used to understand how DEGs function. Furthermore, KEGG pathway and GO enrichment analyses for the DEGs, both up and downregulated from intestinal epithelial tissue of *C. parvum* infected patient samples were performed (Tables S2 and S3). The significant enrichment of up and downregulated DEGs in intestinal epithelial tissue is shown in Figure 3 and Figure 4, respectively. Biological processes (BP) enriched in upregulated DEGs in intestinal epithelial tissue included negative regulation of viral genome replication, negative regulation of viral life cycle, cellular response to type I interferon signaling pathway, cytokine-mediated signaling pathway, regulation of T-helper 2 cell cytokine production, regulation of leukocyte chemotaxis, and positive regulation of leukocyte chemotaxis (Figure 3A). The cladogram results revealed many genes showed expression which were involved in various biological processes such as cytokine-mediated signaling, cellular response to type I interferon, and negative regulation of viral genome replication.

Molecular function analysis suggests that cytokine receptor binding and activity, CXCR chemokine receptor binding, chemokine activity, receptor binding, adenylyl transferase, receptor antagonist activity, and proteasome binding interleukin-6 receptor binding, cAMP-dependent protein kinase regulator activity, and growth factor activity were all abundant (Figure 3B). Changes in cellular component upregulated DEGs were seen in the lipid droplet, perinuclear region of cytoplasm, mitochondrial respiratory chain complex, endocytic vesicle lumen, mitochondrial outer membrane, mitochondrion, cytoplasmic vesicle lumen, fibrillar center, and nuclear inner membrane, among other places (Figure 3C). Biological pathway analysis revealed that the upregulated DEGs were mainly enriched in influenza A, cytokine–cytokine receptor interaction, Hepatitis B and C, TNF, Toll-like receptor, and IL-17 NF-kappa B, chemokine, JAK-STAT signaling pathway, arthritis *Salmonella* infection, rheumatoid and tuberculosis (Figure 3D).

Downregulated DEGs in the intestinal epithelial tissue of *C. parvum* infected patient were enriched in BP, including negative regulation of FGF receptor signaling pathway, chondrocyte development, striated muscle contraction, cellular hyperosmotic response, and negative regulation of BP delayed rectifier potassium channel activity, hyperosmotic response, Wnt signaling etc. (Figure 4A). As for molecular function of the down-regulated DEGs in intestinal epithelial tissue were actin filament binding, proton antiporter, inositol-1,3,4,5-tetrakisphosphate 5-phosphatase activity, etc. (Figure 4B). Changes in cellular component (CC) of downregulated DEGs were mainly enriched endoplasmic reticulum lumen, nucleoplasm part, an integral component of the plasma membrane, golgi sub-compartment, cytoskeleton, nuclear body, nucleolus, mitochondrion, etc. (Figure 4C). Biological pathway analysis revealed the downregulated DEGs are mainly enriched in vitamin A and carotenoid metabolism, wnt signaling, cancer pathway, TLR4 signaling and tolerance, G13 signaling pathway, tryptophan metabolism, differentiation pathway, IL-3, IL-4 signaling pathway (Figure 4D). 

### 3.3. PPI Network and Module Analysis 

**Figure 5:**
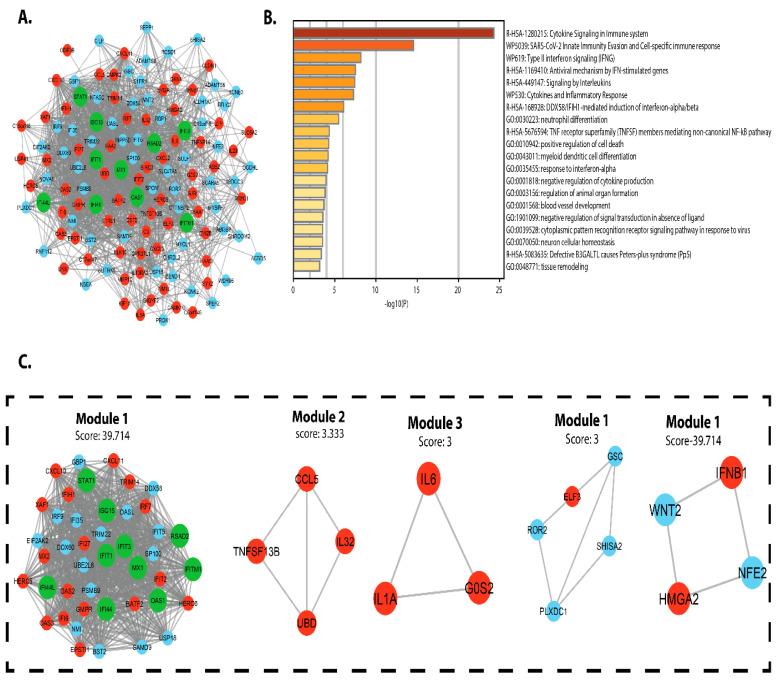
PPI network construction of differential expressed genes in *C. parvum* infected patient’s intestinal epithelial tissue (**A**) PPI network of CPI DEGs red circles represents upregulated DEGs, cyan circle represents downregulated DEGs, and the green circle represents hub genes. (**B**) *p*-values are used to colour a bar graph of enriched terms across all total expressed genes. (**C**) The most significant module was obtained from PPI network.

PPI networks were designed, displayed, and analyzed using Cytoscape software v.3.4.0 and the STRING v.10.5 database. The DEG PPI network was created (Figure 5A), and the essential module was found using Cytoscape (Figure 5B). The topological network properties of DEGs in intestinal epithelial tissue of a *C. parvum* infected patient were analyzed using network analyzer and cytohubba (cytoscope plugin), (Table S3). Metascape was used to perform functional analysis of genes involved in this module [40]. We found that the genes in module 1 were mainly enriched in positive control of cell death, TNFSF members mediating non-canonical NF-kB pathway, neutrophil differentiation, DDX58/IFIH1-mediated activation of interferon-alpha/beta, cytokines, and inflammatory response. Interleukins signaling Type-II interferon signaling (IFNG), SARS-CoV-2 innate immunity evasion and cell-specific immune response, cytokine signaling in the immune system, antiviral mechanism via IFN-stimulated genes.

### 3.4. Biomarker Identification 

**Figure 6:**
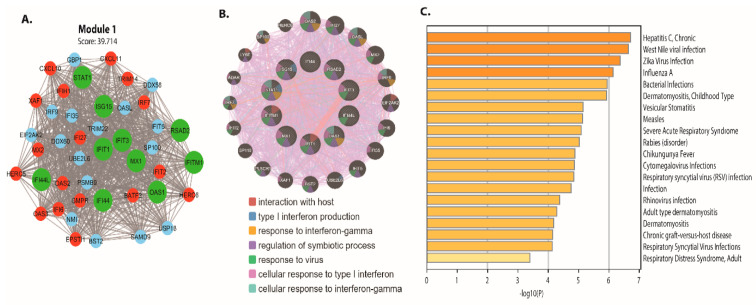
Module 1 PPI network with functional enrichment generated from GeneMANIA. (**A**) A PPI network with 43 nodes and 834 edges yielded the most significant module. (**B**) The module 1 PPI network, which contains 42 genes. Upregulated genes are highlighted in red, downregulated genes are highlighted in cyan, and hub genes are highlighted in green. (**C**) *p*-values are used to color a bar graph of enriched phrases across module 1 genes.

The top ten higher degree (hub genes) in intestinal epithelial tissue of *Cryptosporidium parvum* infected patient DEGs network are identified. The names, abbreviations, degree, and bottleneck for these hub genes are shown in Table 1. The hub genes are ISG15, MX1, IFI44L, STAT1, IFIT1, OAS1, IFIT3, RSAD2, IFITM1, and IFI44. These hub genes were mainly enriched in interaction with the host, type 1 interferon production, response to interferon-gamma Figure 6B. In addition, our study identified four-driver gene biomarkers (IFI44, IFIT3, IFITM1, and MX1) in the network, with many centrality scores (degree and bottleneck). The nodes (proteins or genes) with high centralities are essential to maintain the biological network’s self-organization behavior. We discovered that the majority of the genes are involved in important biological processes such as apoptosis, innate immunity, inflammatory responses, cell proliferation phosphorylation, cell growth, cell cycle, gene expression, cell differentiation, cell signaling, signal transduction, immune responses, and cell apoptosis, among others, using gene enrichment analysis. The most enriched pathways associated with these DEGs were also identified, as shown in Figure 6C. Further research into these infrared genes could lead to a better understanding and prevention of *C. parvum* infection.

### 3.5. Identification of miRNAs Targeting Hub Genes of DEGs Infected by C. parvum

**Figure 7:**
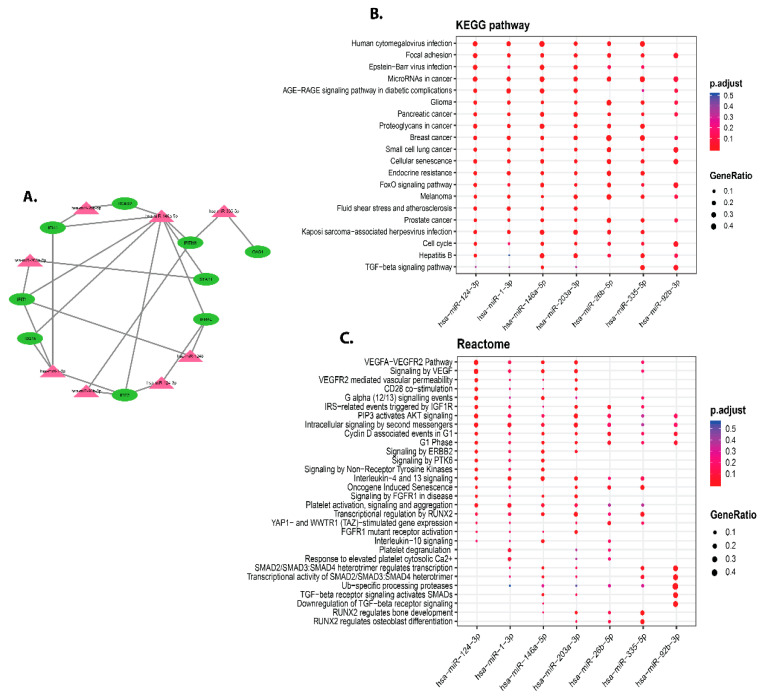
Hub genes and differentially expressed miRNAs are among the MIENTURNET network analysis outcomes. (**A**) MiRNA-hub genes target network visualization, with green triangular circles representing miRNAs and pink circles indicating hub genes miRNAs. (**B**) Based on the enrichment analysis target hub genes for the top ten differentially expressed miRNAs, a dot plot of functional enrichment analysis for the top ten differentially expressed miRNAs. The top 10 differentially expressed miRNAs were chosen for functional enrichment analysis. On the *x*-axis, selected miRNAs are illustrated, while KEGG pathways results are shown on the *y*-axis. The size of the dots reflects the gene ratio, and the colour of the dots indicates the adjusted *p*-value (FDR) (number of miRNA targets found enriched in each category over the number of total genes associated with that category). (**C**) The pathways of the reactome.

MIENTURNET was used to extract the targets of hub gene-miRNAs. The ISG15, MX1, IFI44L, STAT1, IFIT1, OAS1, IFIT3, RSAD2, IFITM1, and IFI44 are the ten hub genes having the highest interactions with miRNAs. Hub genes and miRNAs interacted in networks that were built as shown in Figure 7A. MiR-146a-5p, miR-1-3p, miR-1248, miR-203a-3p, miR-92b-3p, miR-124-3p, and miR-26b-5p are among the major miRNAs that are targeted by hub genes. We found significant correlation between differentially expressed miRNAs and the enrichment of the 08 differentially expressed miRNAs in *parvum infection* (*p* < 0.05 and FDR 0.1). Besides, the 8 hub genes are targeted by miR-146a-5p (RSAD2, STAT1, IFIT1, IFITM1, ISG15, IFI44, IFI44L, IFIT3). Using the KEGG pathway and disease ontology analysis, we assessed the putative functions of the differentially expressed miRNAs. The results underpin how miRNAs were clustered in a variety of pathways, with a focus on the signaling pathway, fluid shear stress, Hepatitis B, cell cycle, kaposi sarcoma-associated herpesvirus infection, atherosclerosis, FoxO signaling pathway, endocrine resistance, and melanoma. Cellular senescence is a term that refers to the ageing of cells. Proteoglycans in cancer, pancreatic cancer, Glioma, AGERAGE signaling pathway in diabetic complications, microRNAs in cancer, Epstein-Barr virus infection, and focal adhesion (Figure 7B). The miRNA is involved in many diseases’ ontology including, cervix carcinoma, interstitial lung disease, pulmonary fibrosis, prostate carcinoma, pancreas adenocarcinoma, diabetic retinopathy, retinal vascular disease, thyroid carcinoma, peripheral nervous system neoplasm, sarcoma, rheumatic disease, breast carcinoma, renal cell carcinoma, bile duct carcinoma, neuroblastoma, autonomic nervous system neoplasm, and autosomal dominant disease. Further, it is involved in many types of cancers, such as large intestine, colorectal, colon, germ cell, thyroid, intestinal, musculoskeletal system, connective tissue, kidney, pancreatic, bile duct, and urinary system cancer. 

## 4. Discussion

Genome-wide gene expression data has been shown to be a reliable resource for identifying molecular pathways underlying human disorders, such as parasite infections. Using in vivo, in vitro, or ex vivo techniques, only a few studies have identified gene expression changes in *C. parvum* infected clinical samples. These investigations, however, have been unable to fully comprehend the role of dysregulated genes at the molecular and network levels [41,42,43]. This study investigated the epithelium’s response to *C. parvum* infection using a series of comprehensive computational analyses. Despite some improvements in Cryptosporidium biology, pathogenicity, and genetic characterization [44,45], no viable cryptosporidiosis control strategies have been discovered. The main problem is that the host-Cryptosporidium interaction mechanism is still poorly understood and comprehended. In the last few decades, researchers have uncovered the nature of host gene non-coding RNA (e.g., lncRNA and miRNA), which has led to new targets and tactics for preventing and treating C. parvum infections in animals and people [46]. In the present study, we systemically investigated the expression profiles of differentiated expressed genes and miRNAs in cells infected with C. parvum using a network theoretical approach.

The study *C. parvum* infected epithelial cells of human have a uniquely diverse gene expression profile, proving new insights into the host’s response. Various studies have shown that infection with *C. parvum* stimulates and suppresses apoptosis in host epithelial cells [47,48,49,50]. RNA molecules are increasingly shown to have a role in various biological processes, including control of gene transcription [51,52]. The two Apicomplexan protozoan parasites, *Plasmodium falciparum,* and *C. parvum*, have been shown to express distinct non-protein-coding RNA genes [53,54,55,56,57]. IFN-inducible genes that produce IFN-induced protein 44 had considerably higher mRNA levels following serotype *Typhimurium* infection (IFI44) [58]. Therefore, it is essential to evaluate the innate immune genes related to type I INF response. Unfortunately, limited information is available to describe immune response expression of genes in infected cells to *C. parvum*. Therefore, the interest of the current study was to find out upregulated and downregulated genes to human type I INFs and epithelial cells infected by *C. parvum*.

In mice, a virulent dam mutant *Salmonella* vs. *WT Salmonella* infection revealed ISG role in host response [59]. Although IFN-/ does not directly activate the MX1 gene, evidence demonstrates that it is involved in the host response to Salmonella infection [59,60]. In uninfected and *Salmonella*-infected macrophages, serine-arginine regulates the expression of numerous gene regulations, with several essential innate immunity genes (Nos2, MX1) relying on several SR/hnRNPs to maintain repression [60]. At the host-parasite interface, *C. parvum* and intestinal epithelial cells interact by transmitting effector molecules from the host cell and the parasite [61]. Studies have shown that some *C. parvum* proteins are transported into host epithelial cells and participate in parasite intracellular growth [61,62]. This has led to studies of selective delivery of low-protein-coding-potential transcripts into infected host epithelia [63]. Moreover, *C. parvum* infection results in the termination of host genes via different mechanisms [20,21], which supports the idea that the infection produces extensive transcriptional gene suppression in infected cells. However, these investigations have been unable to appreciate the molecular and network roles of dysregulated genes fully. The following analysis of intestinal response to *C. parvum* infection investigation includes detailed computational studies. 

Further, TNF and NF-kappa beta signaling pathways were overrepresented in upregulated genes whose biological processes are linked to cytokine–cytokine receptor interaction, TLR4, TNF, IL-17, NF-kappa B, and chemokine signaling pathway. These findings support previous research suggesting that *C. parvum* infection activates the body’s innate immune system by triggering the production of anti-parasitic interferon-gamma cytokine molecules. In addition, Wnt signaling is necessary to repair and regenerate human intestinal epithelial cells when infected with parasites. As a result, *C. parvum* infection may alter cell signaling, obstructing epithelium regeneration and repair.

We discovered ten elevated hub genes implicated in response to *C. parvum* infection (ISG15, MX1, IFI44L, STAT1, IFIT1, OAS1, IFIT3, RSAD2, IFITM1, and IFI44). The overexpression of three biomarkers (IFI44, IFIT3, IFITM1, and MX1), known to directly target the *C. parvum* genome, was verified by protein interaction network analysis DEGs. MiRNAs are known to influence the expression of host genetic components required for infection response. MiRNAs appear to have a posttranscriptional influence on gene expression, affecting various biological processes, including disease onset and progression [64,65,66,67]. MiRNAs are also involved in the regulation of complex parasite–human host interactions. Previous research has looked into the expression of *C. parvum*-induced miRNA in human biliary epithelial cells [64]. The miRNA expression profile of *C. parvum*-infected human intestinal epithelial cells, on the other hand, is unknown. In this study, we discovered that a variety of miRNAs target *C. parvum*-infected intestinal cells. MiR-146b, which binds the TLR4 receptor, regulates the TLR4 signaling pathway [68]. After infection with *C. parvum*, there were no changes in the gene and miRNA expression profiles in the intestinal epithelium. As a result, the goal of this study was to use differential expression and network analysis to examine the intestinal epithelial gene expression profile after *C. parvum* infection. The key miRNAs (MiR-146a-5p, miR-1-3p, miR-1248, miR-203a-3p, miR-92b-3p, miR-124-3p, and miR-26b-5p) targeting multiple host genes presents us a narrow window of novel therapeutic opportunity to target genes in cryptosporidiosis pathophysiology. However, in vitro, ex vivo, and in vivo studies are required to validate the actual role of anti-C. parvum microRNAs in cryptosporidiosis in humans. The primary miRNA (miR-146b) targets many host genes, allowing us a small window of opportunity to target genes involved in *C. parvum* pathogenesis. Anti-*C. parvum* microRNAs must be validated in *C. parvum* infected individuals by in vitro, ex vivo, and in vivo studies.

## 5. Conclusions

The transcriptome of *C. parvum*-infected epithelial cells and healthy controls revealed differentially regulated cellular processes in response to infection. Thus, identifying and quantifying parasite-specific miRNAs and their target miRNAs are critical for a deeper molecular insight in the case of parasitic disease. In addition, the importance of investigating transmission, the likelihood of virulence, and the impact on public health investigations is paramount for every infection. Our findings in the present study would provide novel insights for exploring the control measures for diagntableosis and control of cryptosporidiosis in humans. Therefore, these findings highlight the significance of developing early detection tools and implementing them into clinical practices to identify and treat cryptosporidiosis in humans.

## Figures and Tables

**Table 1:** Top 10 hub genes expressed in intestinal epithelial tissue of *C. parvum* infected patient and its topological properties.

S.No.	Genes	Degree	Gene	BottleNeck
1.	ISG15	355	IFIT3	62
2.	MX1	349	IFITM1	58
3.	IFI44L	291	GBP1	58
4.	STAT1	286	IFI44	56
5.	IFIT1	283	IFIT2	56
6.	OAS1	280	MX1	54
7.	IFIT3	280	TRIM22	54
8.	RSAD2	263	NMI	54
9.	IFITM1	258	IFI27	54
10.	IFI44	251	IFIT5	53

## Data Availability

All relevant data are within the manuscript.

## Supplementary Materials

The following are available online at https://www.mdpi.com/article/10.3390/vaccines9121427/s1, Table S1: List of Up and down regulated differentially expressed genes in *C. parvum*-infected epithelial cells. Table S2: Gene Ontology of Up-regulated DEGs. Table S3: Gene Ontology of down-regulated DEGs.
